# Is it getting better? An analytical method to test trends in health disparities, with tobacco use among sexual minority vs. heterosexual youth as an example

**DOI:** 10.1186/s12939-016-0371-3

**Published:** 2016-05-23

**Authors:** Yuko Homma, Elizabeth Saewyc, Bruno D. Zumbo

**Affiliations:** Mukogawa Women’s University School of Nursing, Hyogo, Japan; Stigma and Resilience Among Vulnerable Youth Centre, University of British Columbia School of Nursing, T201-2211 Wesbrook Mall, Vancouver, BC V6T 2B5 Canada; Measurement Evaluation and Research Methods program, University of British Columbia, Vancouver, BC Canada

**Keywords:** Sexual orientation, Adolescents, School surveys, Tobacco use, Cohort trends, Interaction analysis, Health disparities

## Abstract

**Background:**

Previous studies have documented higher health risks for lesbian, gay, and bisexual youth compared to heterosexual youth. However, none has reported whether the sexual orientation-based gaps have widened, narrowed, or remained unchanged over time. The purpose of this study was to develop a way to test differences in trends between sexual minority and heterosexual youth cohorts in population-based studies, with cigarette smoking as an exemplar.

**Methods:**

We analysed the Minnesota Student Survey of 1998–2010, a repeated, cross-sectional census of adolescent health in grades 9 and 12. Our sample was students with recent sexual experience (*N*s = 17,376–19,617). Sexual orientation was measured by gender of sexual partners in the past 12 months: students with only opposite-gender partner(s) (OPPOS), students with both male and female partners (BOTH), students with only same-gender partner(s) (SAME). We used logistic regressions to examine trends in prevalence of past-month cigarette smoking from 1998 to 2010, separately for each orientation group. We then applied novel interaction analyses to test whether disparities in smoking prevalence between OPPOS and SAME/BOTH changed over time.

**Results:**

Recent smoking rates decreased over time among all orientation groups. BOTH adolescents were more likely than OPPOS adolescents to report past 30-day smoking, but there were no significant differences between SAME adolescents and OPPOS adolescents. Year-by-orientation interactions indicated the gap between BOTH adolescents and OPPOS adolescents widened from 1998 to 2004, then persisted between 2004 and 2010. No significant interaction effects were observed between SAME adolescents and OPPOS adolescents.

**Conclusions:**

All orientation groups had decreasing trends in recent cigarette smoking; however, disparities in smoking rates remain between heterosexual adolescents and bisexual adolescents. These results provide a new method of not just documenting trends within minority groups, but examining whether health equity is improving for them compared to dominant groups.

## Background

A substantial body of population-based evidence in North America, Europe, and elsewhere has documented significant health disparities for lesbian, gay, and bisexual youth (sexual minority or LGB youth) compared to their heterosexual peers [1]. They experience disproportionately higher rates of discrimination and violence exposure [2]; higher prevalence of alcohol and other drug use [3–5]; equal or higher rates of sexual health behaviours, including adolescent pregnancy involvement [6, 7] and for some, greater risk of sexually transmitted infections such as HIV [8, 9]; and higher rates of depression and suicide attempts [10]. Sexual minority youth have also been more likely to report tobacco use than their peers in studies in the USA [4, 11], in the UK [12] and in Canada [13, 14].

Much of the research examining these health disparities links them to stress as a result of stigma, discrimination, and violence targeted toward sexual minority peoples [1]. Yet the past decade has seen sweeping improvements in the social and legal status of sexual minority populations in a number of countries, including changes in human rights law [15] and in legalization of same-sex marriage [16]. There have even been safe school policies enacted to reduce homophobic harassment and bullying among youth in schools [17]. This raises the question: is it getting better? Are these health risks beginning to improve among LGB youth? There are a limited number of regularly repeating population-based adolescent health surveys that include measures of sexual orientation, and have been repeated over a long enough time period to begin to document trends in health and risk behaviours [6, 18]. Declines in health-compromising behaviours or improvements in health outcomes among sexual minority groups, while good news in themselves, cannot tell us whether the disparities between them and heterosexual youth are also improving. For example, tobacco use has been declining among adolescents and adults throughout North America and much of Europe over the past decade [19], so if there are declines in tobacco use among LGB youth, they may be matched by similar declines among heterosexual youth, and thus the gap between them remains. Alternately, tobacco use may be declining at a slower rate for sexual minority youth, widening the gap between them and heterosexual youth, or at a faster rate, narrowing the gap in health equity. Our search of the literature turned up no studies that examine trends over time in health equity between heterosexual and sexual minority people, whether adults or adolescents. Indeed, in searching the literature, we found no articles that directly tested trends in health gaps between any groups, on any health issue.

Part of the reason for this lack of research may be a lack of appropriate analytical methods. Much of the time, health disparities are documented using logistic regressions in order to calculate odds ratios or risk ratios between the dominant majority and the marginalized subgroup. Repeating those analyses separately within multiple cohorts over time and displaying the odds ratios in a table, or graphing them in a figure, which has been used in the past to show persistent disparities [6], is insufficient to accurately evaluate trends in disparities. This is because odds ratios should not be directly compared, not even with confidence intervals, due to underlying heterogeneity that cannot be accounted for in logistic regression models [20]. However, in the past decade, some studies have developed methods to compare odds ratios between independent samples for other purposes, using interaction terms. For example, Altman and Bland [21] recommended using interactions in logistic regression to help determine whether treatment effects differ between two subgroups in intervention studies. These interaction terms produce a ratio of odds ratios within the logistic model. Our question is, could these methods be adapted to test year by orientation group interactions instead, with heterosexual youth as the referent group for orientation, to determine whether the gap is narrowing, widening, or unchanged? If so, this would offer a new method for examining health equity trends among marginalized groups.

Thus, our primary purpose was to adapt these approaches, in order to test trends in health disparities over time between sexual minority and heterosexual youth cohorts in population-based studies. In this analysis, we used disparities in tobacco use among students in the state of Minnesota in the United States of America as the exemplar case to demonstrate the method.

## Methods

### Data

Data were from the Minnesota Student Survey (MSS), a cross-sectional statewide anonymous census of adolescent health administered every 3 years to public school students in grades 9 and 12 in Minnesota. All school districts were invited to participate and school district participation rates were approximately 90 %. Approximately 75,000 students in grades 9 and 12 participated in each year of the survey. The detailed survey procedure has been described elsewhere [22]. We used a weighted, merged data set from 1998 to 2010 for trend analyses, including only school districts that participated in all survey years from 1998 to 2010. The University of British Columbia Behavioural Research Ethics Board approved the study under which these specific analyses were conducted (certificate # H12-00477).

### Sample

As a secondary analysis, we were limited in the measure of sexual orientation to a consistent measure across all the years: our sample only included students who provided responses to two questions: “During the last 12 months, with how many different male partners have you had sexual intercourse?” and “During the last 12 months, with how many different female partners have you had sexual intercourse?” and to gender. Thus our sample consisted of recently sexually active students, grouped by gender of sexual partner into three categories: those who had sex with opposite-gender partner(s) only (OPPOS), those who had sex with same-gender sexual partner(s) only (SAME), and those who had sex with both male and female partners (BOTH). Approximately 30 % of students from the original surveys in each year reported having had sexual intercourse with male and/or female partners in the last 12 months (Ns = 17,376–19,617).

Data were weighted to adjust for differences in student participation rates among school districts in a given year [22]. The weighted sample size is summarized in Table 1, with the percent of grade 12 students noted for each orientation and gender group (the percent of grade 9 students is the inverse of that percent, as only two grades are surveyed).

**Table 1:** Samples Students By Gender of Sexual Partners in the Minnesota Student Surveys

	1998	2001	2004	2007	2010
Male					
OPPOS	7,999	7,347	7,412	7,659	7,832
(Grade 12, %)	(58.7 %)	(63.1 %)	(63.4 %)	(64.9 %)	(66.0 %)
BOTH	1,176	1,008	1,010	1,744	1,632
(Grade 12, %)	(40.6 %)	(46.2 %)	(46.2 %)	(57.7 %)	(55.3 %)
SAME	148	142	172	250	268
(Grade 12, %)	(51.4 %)	(49.3 %)	(48.3 %)	(54.4 %)	(60.4 %)
Female					
OPPOS	8,721	8,210	8,386	8,762	8,869
(Grade 12, %)	(66.8 %)	(69.9 %)	(69.1 %)	(70.7 %)	(71.3 %)
BOTH	367	515	575	658	765
(Grade 12, %)	(47.4 %)	(45.6 %)	(39.8 %)	(48.8 %)	(50.6 %)
SAME	45	56	82	112	138
(Grade 12, %)	(48.9 %)	(44.6 %)	(39.0 %)	(41.1 %)	(58.0 %)

Note. Data were weighted

*OPPOS* Students who had sex with partner(s) of the opposite gender only, *BOTH* Students who had sex with both male and female partners, *SAME* Students who had sex with partner(s) of the same gender only

### Measures

Smoking in the last 30 days was assessed with the item, “During the last 30 days, how frequently have you smoked cigarettes?” Having never smoked a cigarette in the last 30 days was coded as “No” and having smoked less than one cigarette per day or more frequently in the last 30 days as “Yes”.

### Analyses

There were significant gender differences in the prevalence of tobacco use, as well as the prevalence of same-gender or both-gender sexual behaviours (data not shown), so all analyses were stratified by gender.

In order to examine trends over time within each of three orientation groups, we first described the prevalence of cigarette smoking in each of 5 years, separately by sexual orientation. Chi-square test for trend was used to compare 1998, 2004, and 2010 data. Because grade 9 respondents may have completed a subsequent survey when they were in 12^th^ grade, we selected only those 3 years, to ensure sample independence. Likewise, given that there may have been changes over time in the age at which adolescents initiate sexual behaviour or tobacco use, we also conducted logistic regressions, adjusted for grade, to assess changes in the prevalence of cigarette smoking from 1998 to 2004 and from 2004 to 2010 within each orientation group. An odds ratio (OR) greater than 1 indicates an increasing trend, and an OR less than 1 indicates a decreasing trend.

Next, to examine sexual orientation-based disparities in tobacco use within each of the five survey years, we conducted grade-adjusted logistic regressions. The OPPOS group was used as a reference category. Thus, an OR of > 1 indicates that SAME or BOTH students were more likely than OPPOS students to report having smoked a cigarette in the last 30 days. An OR of < 1 indicates a lower likelihood of recent smoking among SAME or BOTH than among OPPOS.

Finally, we examined whether differences in smoking prevalence between OPPOS and SAME and between OPPOS and BOTH widened, narrowed, or stayed the same from 1998 to 2004 and from 2004 to 2010. To do this, we computed interaction terms of sexual orientation and survey year in a logistic regression model that included sexual orientation, survey year, orientation-by-year interaction, and grade, with OPPOS as the reference group for orientation. In this analysis, a statistically significant interaction term suggests that the gap in recent smoking rates between OPPOS and SAME or BOTH has significantly widened or narrowed over time. Basically, this interaction term indicates a ratio of ORs, i.e., a ratio of the OR of smoking by orientation group for a given year (i.e., 1998 or 2010) to the odds of smoking among SAME or BOTH students vs OPPOS students for a reference year (i.e., 2004). An interaction OR was greater than 1 when the OR for a given year was greater than that of the reference year, whereas an interaction OR was smaller than 1 when the OR for a year was smaller than the OR of the reference year. To interpret these interaction ratios of ratios, however, it is important to pay attention to the main effect OR in the model as well: when both the OR for a given year and OR for a reference year are greater than 1, an interaction OR greater than 1 suggests the sexual orientation-based disparity in a year was larger than in the reference year (i.e., the gap is widening), and an interaction OR less than 1 suggests the orientation-based difference in a year was smaller than in a reference year (i.e., the gap is narrowing). In contrast, when both the original year ORs are smaller than 1, an interaction OR greater than 1 suggests the inverse: that the orientation-based difference in a given year is smaller than in a reference year (gap is narrowing), whereas an interaction OR less than 1 suggests the orientation-based difference in a given year is greater than in the reference year (the gap is widening). To determine whether the gap has widened or narrowed, one needs to refer to the OR for the sexual orientation-based difference in a given year and in a reference year along with the interaction OR. See Table 2 for a summary of interpreting odds ratios for the interaction terms.

**Table 2:** Interpretation of Odds Ratios for Analyses of Trends in Disparities

	Original ORs^a^	ORs for interaction terms	Sexual orientation-based disparities
Year 1998 or 2010	>1	>1	Widening
Year 2004 (reference)		<1	Narrowing
Year 1998 or 2010	<1	>1	Narrowing
Year 2004 (reference)		<1	Widening

Note. *OR* Odds ratio

^a^ORs from logistic regression models that examine sexual orientation-based disparities in tobacco use within each of the 3 years (1998, 2004, and 2010)

A question may arise, why are these complex interaction terms needed for testing trends in disparities? Couldn’t we just compare the trends in the prevalence (i.e., the percents) between heterosexual and sexual minority youth, rather than comparing trends in the odds ratios or adjusted odds ratios? Indeed, Asada [23] notes the importance of examining absolute measures of inequalities that take into account the starting point, or the absolute level of the health issue in each group, when comparing populations. Asada asserts that by calculating the absolute change in each group by subtracting the last year percent from first year percent, the absolute size of the change can hint at the trend in the inequality, i.e., the inequality is greater when the dominant group improves more than the minority group, and it is smaller when the minority group improves more than the minority group. The problem with directly comparing trends in prevalence between heterosexual and sexual minority youth as an absolute difference in percentage points is that these results may be somewhat misleading, because simple percents do not account for age differences in the two samples that can provide a competing explanation for the disparities. Because sexual orientation is a developmental task of adolescence, and development itself has a wide range of normal variation in timing, not all young people go through puberty, develop attractions, or begin romantic or sexual relationships at the same age, and if the attractions or identity are stigmatized, it tends to be longer before young people publicly disclose that, even if they act on the knowledge earlier [1]. Thus, in secondary school population surveys throughout the world, researchers have generally noted significant age differences between heterosexual and sexual minority youth, no matter how sexual orientation is measured [1, 24, 25], although those age differences are not always in the same direction. For example, youth engaging in same-gender or both-gender sexual behaviour tend to report that at slightly younger ages than those engaging in opposite-sex sexual behaviours [6], which appears to be due in part to their greater risk for being targeted for sexual abuse during early adolescence [2, 8].

In contrast, among those with same-sex attractions, stigma may delay their recognition of and public disclosure of such attractions [1]. And when it comes to identity labels, because of our heteronormative society, many adolescents who have not yet developed attractions or engaged in sexual behaviour identify as the default heterosexual, and it is only later during adolescence that they identify as lesbian, gay, or bisexual [26]. Thus, in population-based surveys, the average age of sexual minority youth tends to be different from the average age among heterosexual youth, but may be older or younger, depending on whether the sample is limited to sexually experienced youth, or all youth. And since smoking itself has a maturational trend (older youth are more likely to smoke than younger adolescents), unless the analyses controls for age (or grade in studies where the sample is only within discrete grades), the prevalences for heterosexual and sexual minority youth are not truly comparable, they're confounded by demographic differences in age. Therefore, age- or grade- adjusted regressions are potentially more appropriate comparisons to use in order to identify trends in disparities.

Documenting the strength of disparities between groups, and testing trends in disparities, however, are only part of the information needed to understand and address health equity issues. Odds ratios, even adjusted odds, are a measure of the relative strength of the disparity between two groups, but provide no information about the scope or magnitude of the problem. For example, an odds ratio of 2.0 could describe a disparity in tobacco use between two groups when the difference is 1.5 % vs. 3.0 %, or when it is 45 % vs. 90 %, but most people would find the second disparity far more urgent a concern. As well, the size of the percentage change within a group over time may provide a clue as to which group’s improvement or decline might be driving the widening or narrowing (or unchanged) gap between the groups. Therefore, it is important to report the prevalence to give readers a sense of the size of the issue, while using age-adjusted regressions to describe the strength of the disparity between both groups as an adjusted odds ratio, and to test the trends in the disparity using the ratio of adjusted odds ratios.

## Results and discussion

### Trends in past-month cigarette smoking within each orientation group

As shown in Table 3, recent cigarette smoking rates have generally declined over time within all sexual orientation groups. Past 30-day smoking rates have decreased from 1998 to 2004 among all male groups, but in 2010, smoking in the last 30 days prevalence decreased compared to 2004 among OPPOS boys and BOTH boys, but not among SAME boys. Among girls, significant declines from 1998 to 2004 were observed among OPPOS and BOTH but not among SAME, however, all female groups in 2010 were less likely than their 2004 counterparts to report smoking cigarettes in the last 30 days. The absolute differences in declining prevalence among the OPPOS boys (26.8 points) and girls (34.4 points) appears to be larger than it is among BOTH and SAME students (24.4 for BOTH and 21.1 for SAME boys; 25.8 for BOTH and 21.5 for SAME girls), however, it is important to remember these are not adjusted for age.

**Table 3:** Trends in Prevalence of Recent Cigarette Smoking Across Years, within Sexual Orientation Groups

	Ever smoked cigarettes in the last 30 days (%)						Trend 1998 – 2004^b^	Trend 2004 – 2010^c^
	1998	2001	2004	2007	2010	*p*-value^a^	OR^d^ (95% CI)	OR^d^ (95% CI)
Male								
OPPOS	56.3	47.0	38.5	34.1	29.5	< .001	**0.48 (0.45, 0.51)**	**0.66 (0.62, 0.71)**
BOTH	63.3	54.6	50.9	40.1	38.9	< .001	**0.59 (0.49, 0.70)**	**0.59 (0.50, 0.70)**
SAME	52.1	35.0	34.5	35.7	31.0	< .001	**0.48 (0.30, 0.76)**	0.83 (0.54, 1.25)
Female								
OPPOS	63.9	52.9	44.6	36.3	29.5	< .001	**0.46 (0.43, 0.48)**	**0.52 (0.49, 0.56)**
BOTH	75.8	67.3	66.7	56.9	50.0	< .001	**0.63 (0.46, 0.85)**	**0.51 (0.40, 0.64)**
SAME	55.8	40.0	48.1	31.7	34.3	.006	0.74 (0.35, 1.59)	**0.53 (0.30, 0.96)**

Note. Data were weighted. OR in bold indicates *p* < .05

^a^Chi-square test for trend

^b^Reference year = 1998

^c^Reference year = 2004

^d^Adjusted for grade

*OPPOS* Students who had sex with partner(s) of the opposite gender only, *BOTH* Students who had sex with both male and female partners, *SAME* Students who had sex with partner(s) of the same gender only, *OR* Odds ratio, *CI* Confidence interval

### Disparities in smoking between sexual minority and heterosexual groups in each year

Table 4 presents odds ratios (ORs) adjusted for grade to document disparities in recent cigarette smoking between sexual minority and heterosexual students within each survey year. BOTH and SAME groups were compared to the reference group of OPPOS. Overall, As shown in Table 3, BOTH boys and BOTH girls were more likely than their OPPOS peers to report having smoked a cigarette in the last 30 days. On the other hand, there were no significant differences in recent smoking between SAME groups and OPPOS groups, except for boys in 2001, when SAME boys were significantly less likely than OPPOS boys to have smoked a cigarette in the last 30 days. There were no grade-adjusted significant differences between SAME girls and OPPOS girls in recent tobacco use in any year.

**Table 4:** Sexual Orientation Disparities in Recent Cigarette Smoking, within Year: Odds Ratios^a^ and 95 % Confidence Intervals

	1998	2001	2004	2007	2010
Male					
OPPOS	ref	ref	ref	ref	ref
BOTH	**1.36 (1.19, 1.55)**	**1.43 (1.25, 1.64)**	**1.73 (1.51, 1.99)**	**1.33 (1.19, 1.48)**	**1.60 (1.43, 1.80)**
SAME	0.86 (0.62, 1.19)	**0.63 (0.44, 0.90)**	0.87 (0.63, 1.20)	1.12 (0.86, 1.47)	1.10 (0.84, 1.44)
Female					
OPPOS	ref	ref	ref	ref	ref
BOTH	**1.66 (1.30, 2.13)**	**1.76 (1.45, 2.14)**	**2.36 (1.96, 2.84)**	**2.27 (1.92, 2.68)**	**2.33 (2.00, 2.71)**
SAME	0.67 (0.36, 1.24)	0.56 (0.33, 0.97)	1.08 (0.69, 1.70)	0.79 (0.52, 1.20)	1.24 (0.87, 1.78)

Note. Data were weighted. 95 % confidence intervals are in parentheses, Odds ratios in bold indicate *p* < .05

^a^Adjusted for grade

*OPPOS* Students who had sex in past year with partner(s) of the opposite gender only, *BOTH* Students who had sex in past year with both male and female partners, *SAME* Students who had sex in past year with partner(s) of the same gender only, *ref* Reference group

### Trends in sexual orientation disparities in smoking: Is it getting better?

All sexual orientation groups had declining trends in last 30-day cigarette smoking between 1998 and 2010. However, BOTH boys and girls continued reporting higher adjusted odds of smoking than their OPPOS peers. We then examined whether the differences in smoking rates by sexual orientation have been smaller or larger from 1998 to 2004 and from 2004 to 2010. ORs for interaction terms of survey year and sexual orientation are presented in Table 5. The statistically significant interaction between BOTH and year 1998 for last 30-day smoking among boys and girls indicates that the gap between BOTH and OPPOS has widened from 1998 to 2004, then persisted between 2004 and 2010. The odds ratios were from 1.36 to 1.73 among BOTH boys and from 1.66 to 2.36 among BOTH girls (Table 3). There were no significant interaction effects among SAME groups compared to OPPOS groups, and no disparities in tobacco use. The widening gap between BOTH and OPPOS groups may be due to steeper declines in tobacco use among OPPOS groups than among BOTH groups.

**Table 5:** Trends in Disparities in Recent Cigarette Smoking: Interactions Between Sexual Orientation and Year

	Male	Female
	OR^a^ (95% CI)	OR^a^ (95% CI)
OPPOS by Year 2004	ref	ref
BOTH by Year 1998	**0.81 (0.67, 0.98)**	**0.73 (0.54, 0.99)**
BOTH by Year 2010	0.91 (0.76, 1.08)	0.98 (0.77, 1.24)
SAME by Year 1998	1.00 (0.63, 1.58)	0.65 (0.30, 1.38)
SAME by Year 2010	1.25 (0.82, 1.91)	1.15 (0.65, 2.04)

Note. Data were weighted. Odd ratio in bold indicates *p* < .05

^a^The model included sexual orientation, survey year, and grade along with orientation-by-year interactions

*OR* Odds ratio, *CI* Confidence interval, *OPPOS* Students who had sex with partner(s) of the opposite gender only, *BOTH* Students who had sex with both male and female partners, *SAME* Students who had sex with partner(s) of the same gender only, *ref* Reference group

In this paper, we have documented trends in recent cigarette use among sexually active heterosexual and sexual minority adolescents in Minnesota schools between 1998 and 2010. As demonstrated in recent studies of tobacco use among general populations of adolescents in high-income countries, we found steady declines in tobacco use within each orientation group across the years. In each year of the survey, we found disparities in past-month cigarette smoking between youth with bisexual vs. heterosexual sexual behaviours, which are similar to findings from other cross-sectional studies. In contrast to other studies, however, we did not find disparities between youth who report only same-gender sexual behaviour and those with heterosexual behaviour. This may be partly explained by differences in how sexual orientation is measured in the Minnesota Student Survey as compared to other studies, as some studies combine lesbian, gay and bisexual adolescents, thus potentially masking differences within sexual minority groups. Likewise, studies that use attraction or identity as the sexual orientation measure, although generally considered a more valid measure than behaviour alone [1, 26], may end up combining youth with solely same-gender sexual partners and those with both-gender sexual partners, because attraction, identity and actual sexual behaviour are not necessarily concordant among adolescents [9, 24, 25]. However, other studies have documented higher levels of health challenges among bisexual youth and adults, in part because of lack of acceptance in gay and lesbian communities as well as heterosexual communities, and these disparities in tobacco use for youth with bisexual behaviour are of concern.

We also demonstrated a novel technique for testing whether these disparities between sexual minority and heterosexual youth tobacco use were significantly increasing, declining, or unchanged, and found that the gap between bisexual and heterosexual youth’s tobacco use widened between 1998 and 2004 for both boys and girls reporting both-gender sexual partners, and these disparities continued between 2004 and 2010. This analysis suggests that while population-wide interventions to reduce tobacco initiation among adolescents is having some effect on sexual minority youth, among those with bisexual behaviour, the interventions are not as effective as on those with exclusively monosexual behaviour, whether opposite-sex only or same-sex only. The reasons for such widening disparities over the past decade are not clear, but one possible explanation is that bisexual invisibility, or biphobia in both mainstream community and LGBTQ communities increase the minority stress for bisexual adolescents, and reduce their social supports. Thus, additional targeted interventions may be needed, either to reduce biphobia and stigma-related stress or improve social supports, in order to further reduce the prevalence of tobacco use among bisexual adolescents.

### Strengths and limitations

As with all studies, this analysis has both strengths and limitations. A key strength is the use of regularly repeated large-scale population surveys of adolescents in school, with one of the few such surveys that covers more than a decade and includes a measure of sexual orientation, with large enough sample sizes to disaggregate those with same-gender only and both-gender sexual partners. As well, the use of an established statistical technique, although applying it for a novel analytical purpose, is a clear innovation that may be useful for measuring trends in health equity for sexual minority youth and potentially other marginalized groups.

At the same time, it must be acknowledged that the measure of sexual orientation itself is sub-optimal. Since the majority of adolescents in North America are not sexually active, a measure of sexual orientation that relies on sexual behaviours will inevitably exclude a significant proportion of the population, either heterosexual or sexual minority youth. Sexual orientation has been more accurately assessed among adolescents as attractions or identity labels [26] as there is noted discordance between attractions/identity and actual sexual behaviour [9, 24, 25]. As well, focusing on the gender of past year sexual partners may misclassify youth with only one sexual partner in the past year, whether same-gender or other-gender; if any previous partners in earlier years were of a different gender, they would more accurately belong in the both-gender category. These trend analyses should be replicated with other population-based surveys that also assess sexual orientation through attraction or identity measures, so that youth who are not sexually experienced are included in the study. These results are from a single state in the US, Minnesota; different environments, laws, culture and history in other regions of the US might influence trends in health disparities for sexual minority youth in different directions than we found in Minnesota. Similarly, trends in disparities may look different in Canada, or in other countries outside of North America; these methods should be used where possible to document whether disparities are getting better in different regions globally, wherever it is feasible given existing population surveys. Finally, as a study of youth in school, the results cannot be generalized to youth who are not attending school.

## Conclusions

This is among the first few studies to document trends in health and risk behaviours among sexual minority adolescents, and to our knowledge, is the first to actually test trends in health disparities for them. The results show the importance of not only documenting trends among sexual minority populations, but also documenting whether health equity is improving for them compared to dominant groups. This information can help guide policies, practices, and resource allocation to reduce health inequities.

This innovative method of testing trends in health disparities, however, to see if the gap is narrowing, widening, or unchanged, has relevance for health equity beyond that of sexual minority people. The technique could be applied within national, regional, and local population studies to evaluate trends in health disparities among other groups who experience significantly higher health risks or poorer health outcomes as a result of social marginalization; for example, disparities between ethnocultural minority groups and the dominant groups in societies, or between low-income groups and those with higher socio-economic status. It may not be enough to track changes in health outcomes for Indigenous populations, or for groups of children in foster care, for example, without placing these trends in the context of the wider community’s trends in health improvements. This new approach to testing the trend in disparities is one way to take context into account, and will provide important information to guide health equity initiatives for marginalized groups.

## Abbreviations

BOTH, adolescents who had sex with both male and female partners; LGB, lesbian, gay, and bisexual; OPPOS, adolescents who had sex with opposite-gender partner(s) only; SAME, adolescents who had sex with same-gender partner(s) only.
